# Circulating microRNAs in Early Breast Cancer Patients and Its Association With Lymph Node Metastases

**DOI:** 10.3389/fonc.2021.627811

**Published:** 2021-08-26

**Authors:** Daniel Escuin, Laura López-Vilaró, Josefina Mora, Olga Bell, Antonio Moral, Ignacio Pérez, Cristina Arqueros, Bárbara García-Valdecasas, Teresa Ramón y Cajal, Enrique Lerma, Agustí Barnadas

**Affiliations:** ^1^ Clinical Oncology Research Group, Institut d'Investigacions Biomédiques Sant Pau (IIB-Sant Pau), Barcelona, Spain; ^2^ Department of Pathology, Hospital de la Santa Creu i Sant Pau, Barcelona, Spain; ^3^ Department of Clinical Biochemistry, Hospital de la Santa Creu i Sant Pau, Barcelona, Spain; ^4^ Department of General Surgery, Hospital de la Santa Creu i Sant Pau, Barcelona, Spain; ^5^ School of Medicine, Universitat Autónoma de Barcelona (UAB), Cerdanyola del Vallès, Spain; ^6^ Department of Medical Oncology, Hospital de la Santa Creu i Sant Pau, Barcelona, Spain; ^7^ Department of Gynecology, Hospital de la Santa Creu i Sant Pau, Barcelona, Spain; ^8^ Centro de Investigación Biomédica en Red Cáncer (CIBERONC), Madrid, Spain

**Keywords:** circulating microRNAs, breast cancer, lymph node, metastasis, surrogate biomarker

## Abstract

MicroRNAs have emerged as important regulators of the metastatic process. In addition, circulating miRNAs appear to be surprisingly stable in peripheral blood making them ideal noninvasive biomarkers for disease diagnosis. Here, we performed a proof-of-principle study to investigate the expression profile of circulating miRNAs and their association with the metastatic lymph node status in early breast cancer patients. Sentinel lymph node status was detected by one-step nucleic acid (OSNA) analysis. We performed RNA-sequencing in 16 plasma samples and validated the results by qPCR. Gene Ontology term enrichment and KEGG pathway analyses were carried out using DAVID tools. We found16 differentially expressed miRNAs (q < 0.01) in patients with positive SLNs. Fourteen miRNAs were down-regulated (miR-339-5p, miR-133a-3p, miR-326, miR-331-3p, miR-369-3p, miR-328-3p, miR-26a-3p, miR-139-3p, miR-493-3p, miR-664a-5p, miR-146a-5p, miR-323b-3p, miR-1307-3p and miR-423-3p) and 2 were up-regulated (miR-101-3pand miR-144-3p). Hierarchical clustering using differentially expressed miRNAs clearly distinguished patients according to their lymph node status. Gene ontology analysis showed a significant enrichment of biological processes associated with the regulation of the epithelial mesenchymal transition, cell proliferation and transcriptional regulation. Our results suggest the potential role of several circulating miRNAs as surrogate markers of lymph node metastases in early breast cancer patients. Further validation in a larger cohort of patients will be necessary to confirm our results.

## Introduction

Breast cancer remains a common disease worldwide and the second cause of cancer death in the US (1). Early diagnosis, improvements in treatment and early onset of therapy are important factors determining the prognosis and management of patients with breast cancer. Various factors such as early age at menarche, late age at first birth and late age at menopause are related to breast cancer risk. However, lymph node (LN) affection remains the most important prognosis factor in breast cancer (2). There are a number of factors associated with metastases to the LN, including tumor size, presence of lymphovascular invasion, poor histological grade and age (3, 4). Nevertheless, for a significant number of early-breast cancer patients it is unclear whom will develop metastases. For instance, about 13% of patients with favorable prognostic factors at diagnose will develop metastasis and the percentage increases to 20-30% for LN-negative patients. In contrast, 20-30% of LN-positive patients will never metastasize (5), therefore it is unclear whether distant metastases arise in a sequential manner from LN metastases or in parallel through the blood stream and whether other factors such interactions between the tumor and the stroma favor locoregional metastases (6).

Most women diagnosed with breast cancer are initially treated with surgery to remove the tumor and to determine the presence of metastases in the sentinel LNs (SLNs). This is currently the recommended procedure for axillary staging of early breast cancer. Our institution use the one-step nucleic amplification (OSNA) assay (7) to accurately measure total metastatic volume in the SLN (8), as an alternative to intraoperative microscopy-based pathological assessment of the SLN. The OSNA assay is a rapid molecular detection of SLN metastasis based on the semi-quantification of cytokeratin 19 (CK19) mRNA copy numbers (7). Thus, only patients diagnosed with more than two macrometastatic SLN are further treated with axillary lymph node dissection (ALND) (9), the golden standard procedure for invasive breast cancer. However, ALND has been questioned in recent years because of inherent morbidity following the procedure without directly contributing to survival in primary breast cancer patients (10–12) and the recognition that not all patients with nodal disease may require extensive axillary surgery (13).

Elucidation of breast cancer’s molecular biological features have had a dramatic effect on how patients are diagnosed and treated. However, effective management of breast cancer is still difficult because of the lack of sensitive and specific biomarkers for early detection and for diseases monitoring. Accumulating evidence in the last years has highlighted the potential use of peripheral blood circulating nucleic acids in breast cancer diagnosis, prognosis and for monitoring response to anticancer therapy. Among these, circulating microRNAs (miRNAs) are increasingly recognized as a promising non-invasive biomarker, given the ease with which miRNAs can be isolated and their structural stability under different conditions of sample processing and isolation (14–16).

MicroRNAs (miRNAs) are a small (19-25 nt) non-coding RNAs, expressed in a wide variety of organisms and highly conserved across species (17). MiRNAs regulate the expression of target genes by binding to complementary regions of messenger transcripts to repress their translation or regulate their degradation. MiRNAs are now recognized as novel post-transcriptional regulators targeting over 30% of the human genome (18). The overall emerging picture is that of a complex regulation level of gene expression, in which a single miRNA may control hundreds of targets (19). Many cellular pathways are affected by the regulatory function of miRNAs and several human pathologies, including cancers, have been associated with misregulation of the miRNAs (16) and their metastases (20). Numerous studies have identified widespread alterations in the expression of miRNAs related to human neoplasias. In breast cancer, analysis of miRNA expression classified the different breast cancer molecular subtypes and correlated these with various clinicopathological factors and numerous miRNAs have been shown to play a pivotal role in various steps of the metastatic process. In addition, circulating miRNAs are emerging as prognostic factors in breast cancer (14), but few studies have correlated their expression with the LN status, the occurrence of distant metastases and breast cancer recurrence. These studies have analyzed the expression of specific circulating miRNAs by qPCR and have shown promising results (21–24).

Herein, we sought to examine the miRNA content in plasma samples from early breast cancer patients with known SLN and axillary LN metastatic status. We designed a proof-of-principle study to profile the expression of miRNAs by RNA-sequencing using preoperative peripheral blood from patients with early breast cancer who were not previously treated. Our results are preliminary but support the hypothesis of the existence of a differential miRNA expression profile in the peripheral blood from breast cancer patients associated with the LN status of their tumors. Our data highlights the potential use of circulating miRNAs as surrogate markers of locoregional metastases in breast cancer. Further studies in a larger number of samples are warranted.

## Materials and Methods

### Patients

We studied 16 patients with early breast cancer treated with surgery and diagnosed for positive SLNs. All patients had confirmed diagnosis based on histopathology of tumor biopsy. All tumors were invasive ductal carcinomas (IDCs) with or without *in situ* component. In 2 cases, tumors were mixed and show presence of invasive lobular carcinoma (ILC) component. Intraoperative SLN were evaluated using the OSNA assay (7). None of the patients had prior treatment with surgery, chemotherapy or radiation. All patients were hormone receptor (HR) positive, HER2 negative. We collected the following clinical and pathological parameters: age, menopausal status, personal and familiar disease precedents and clinical follow-up, tumor stage was be determined according to the AJCC/UICC system (25), histological grade was determined using the Elston-Ellis grading system (26), histology (ductal, lobular, special types), presence of associated ductal or lobular carcinoma *in situ*, presence of vascular and lymphatic invasion, tumor infiltrating lymphocytes, type of invasion (expansive/infiltrating), tumor multifocality, tumor necrosis; proliferation of non-tumoral tissue (ductal hyperplasia, atypical ductal/lobular hyperplasia).

### Blood Processing and Isolation of Plasma

Human plasma samples were collected prospectively from early breast cancer patients who have not received any previous treatment. Peripheral blood was withdrawn before surgery. Approximately, 10-15ml of peripheral blood was collected for plasma processing in EDTA tubes. Plasma tubes were processed within 2 hours of collection and spun at 1200xg for 10 minutes. Plasma was aliquot in 1.5 ml tubes and stored at -80C until further processing. All plasma samples used in this study were inspected for absence of hemolysis as previously described (27). Briefly, the hemolysis score (HS) was determined by ultraviolet-visible (UV-Vis) absorbance measurements using a NanoDrop^®^ 2000 Spectrophotometer (Thermo Scientific, Barrington, IL, USA). Measurements were performed by applying 2 µl of plasma on the micro-volume pedestal after centrifugation at 1000 × g for 5 min at 4°C and using saline (PBS) as a blank. In addition, monitoring of hemolysis was conducted by qPCR for all samples by comparing the level of a highly expressed miRNA in red blood cells (hsa-miR-451a) with a miRNA unaffected by hemolysis (hsa-miR-23a-3p) as previously described (28). Samples with a ΔCt > 7 were discarded for further analyses.

### RNA Isolation NGS Library Preparation and Next Generation Sequencing

RNA was isolated from 300μl of plasma samples with the miRNeasy serum/plasma advanced kit (Qiagen, Cat No/ID: 217204) according to the manufacturer’s instructions. A range of spike-ins were added to the plasma samples prior to RNA isolation. A quality check was performed by qPCR previous to sequencing the samples. Sixteen samples were selected to perform NGS, including 12 positive SLNs (n = 6 macrometastasis and n= 6 micrometastasis) and 4 negative SLNs. Five μl of total RNA was used to construct the NGS libraries using the QIAseqmiRNA Library Kit (Qiagen, Cat. No: 331505). Briefly, after ligation of 3’ and 5’ adapters and Unique Molecular Identifier (UMIs) to miRNAs, complementary DNA libraries were constructed by reverse transcription followed by 22 cycles of PCR amplification and cDNA cleaned up using QMN beads. A library preparation quality check was performed using either Bioanalyzer 2100 (Agilent) or TapeStation 4200 (Agilent). Based on quality of the inserts and the concentration measurements, libraries were pooled in equimolar ratios and quantified using the qPCR ExiSEQ LNA™ Quant kit (Exiqon). The library pools were sequenced with a NextSeq500 platform (Illumina) using sequence runs of 75nt single-end reads with an average number of 10 million reads/sample. Raw data was demultiplexed and FASTQ files were generated using the bcl2fastq 2.18.0.12 software (Illumina) and files were checked using the FastQC tool (http://www.bioinformatics.babraham.ac.uk/projects/fastqc/).

### Genome Annotation and Quantification of miRNAs

Genome annotation was performed using the Exiqon/Xplore RNA pipeline. Following sequencing, Cutadapt (1.9.1) (29) was used to trimmed adaptor sequences. A quality check (QC) was performed to ensure Q-scores >30 (>99.9% correct) of our data (30). Reads with correct length were analyzed for the presence of UMIs using Cutadapt (1.9.1) and then collapsed by UMIs into FASTQ files. This approach eliminates library amplification bias and allows for true identification of the miRNAs. Bowtie2 software (2.2.6) was used for mapping the reads. The mapping criterion for aligning reads to spike-ins, abundant sequences and miRBase_20 was for reads to have perfect match to the reference sequences. To map the genome, one mismatch was allowed in the first 32 bases of the read. Small insertions and deletions (INDELs) were not allowed. The resulting sequences were annotated using the human assembly GRCh37 and the miRBase_20 database. IsomiR analysis was performed individually for each sample based on the occurrence of count variants for each detected miRNA. Read variants were merged onto a single count file with a consistent nomenclature across samples. Only isomiRs present at a level of 5% of total reads for a specific miRNA were retained. Transcripts per million (TPM) was used as a normalization procedure to correct for differences in sequencing depth and to quantified each RNA species.

### Differential Expression Analysis

Differential expression analysis was performed using the EdgeR statistical software package (Bioconductor, http://www.bioconductor.org/). The analysis was performed using the trimmed mean of M-values normalization method (TMM) (31), based on the log-fold and absolute gene-wise changes in expression between samples. The TMM normalization compensates for sample specific effects caused by the variation in library size/sequencing depth between samples and also compensates for under- or over-sampling effects by trimming and scaling factors that minimize log fold changes between samples across the majority of the miRNAs. The isomiR analysis was done using Exiqon in-house scripts (exq_ngs_mircount). Predicted miRNAs analysis was performed based on the read count distribution using the exiqon_ngs_mirpred in house script and the secondary structure prediction according to the miRPara classification score (32). Volcano plots were constructed using R programming (33) by plotting the p value (-log10) on the y-axis and the expression fold change between the two experimental groups on the x-axis.

### Principal Component Analysis and Heat Map and Unsupervised Clustering

Principal component analysis (PCA) was performed using R programming and TMM-normalized values as input. The same input was used to generate a heatmap and unsupervised hierarchical clustering by samples and gene expression profile with R scripts (33). We selected the top 50 miRNAs with the largest coefficient of variation (% CV) across all samples to obtain a cluster of samples. The data was normalized to TMM and converted to log2 scale.

### Gene Ontology Enrichment Analysis

Gene Ontology (GO) analyses (34, 35) were done with R TopGO package with experimentally verified targets of significantly differentially expressed miRNAs as input. Two different statistical tests were used and compared. First, a standard Fisher’s test was used to investigate enrichment of terms between groups. Second, the Elim method (36) was used to incorporate the topology of the GO network and to compensate for local dependencies between GO that could mask significant GO terms. Comparisons from these two methods were used to highlight relevant GO terms.

### Quantitative Real-Time RT–PCR Analysis

Quantitative real-time RT–PCR analysis was done with an ABI Prism 7500 Sequence Detection System using the miRCURY LNA™ Universal RT cDNA Synthesis Kit (Exiqon). The cDNA was diluted 50X and assayed in 10 µl PCR reactions according to the protocol for the miRCURY LNA™ Universal RT microRNA PCR System (Exiqon A/S); each microRNA was assayed twice by qPCR on the plasma Focus microRNA PCR panel. A no-template control (NTC) of water was purified with the samples and profiled like the samples. Analysis of the data was performed using the relative miRNA expression according to the comparative Ct (ΔΔCt) method using negative metastatic samples as reference. We used the geNorm (37) or the Normfinder algorithm (38) to select the best combination of two reference genes. Data from multiples plates were normalized using UniSp3 spike-in (Exiqon) as interplate calibrators.

### Statistics

Differentially expressed miRNAs from RNA-sequencing data were detected by an exact test based on conditional maximum likelihood (CML) included in the R Bioconductor package edgeR (39). *P* values from RNA-sequencing were corrected (q-values) for multiple testing using the Benjamini-Hochberg procedure (40). A false discovery rate (FDR) q < 0.05 was considered significant. In all group comparisons missing expression values were treated as zero. Differences in total numbers of miRNAs between groups were analyzed by two-sided parametric t-tests. For analysis of clinicopathological parameters, quantitative variables between groups were compared using the Student’s T-test and qualitative variables were compared using the X_2_ or Fisher exact tests. A two-sided p-value ≤ 0.05 was considered significant.

## Results

A total of 25 patients were included in this study. However, only samples from 16 patients passed the pre-RNA-sequencing quality check (QC). The main clinicopathological characteristics of the patients are described in **Table 1**. A total of 12 (75%) patients had SLN-positive tumors (76%), of which 6 were OSNA-diagnosed as micrometastasis (38%) and 6 as macrometastasis (38%).

**Table 1:** Basic patient and tumor characteristics.

Variable		N (%)
Patients	Plasma	16 (100)
Age, years	Mean +SD	63 ± 13
	Median (range)	63 (46 - 89)
Tumor status	T1	10 (62)
	T2	2 (13)
	T3	4 (24)
Node status	Negative	4 (25)
	Micrometastasis	6 (38)
	Macrometastasis	6 (38)
Axillary Lymph Node Status	Negative	3 (50)
	Positive	3 (50)
Tumor grade	I	5 (31)
	II	9 (56)
	III	2 (13)
Estrogen receptor	Negative	0 (0)
	Positive	16 (100)
Progesterone receptor	Negative	1 (6)
	Positive	15 (94)
HER2 status	Negative	16 (100)
	Positive	0 (0)
Ki67	< 20%	15 (94)
	> 20%	1 (6)
Surgery	Mastectomy	5 (31)
	Lumpectomy	11 (69)
Lymphovascular invasion	Negative	12 (75)
	Positive	4 (25)

**Figure 1:**
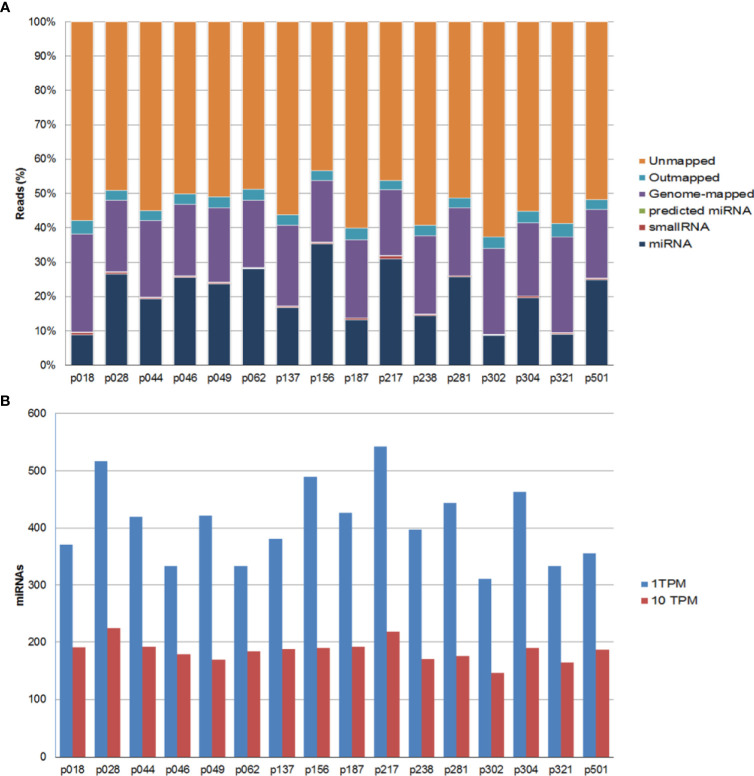
Summary of the mapping results for all samples. **(A)** Percentage of sequencing reads for each sample. Reads are classified as miRNAs, small RNAs, genome-mapped, outmapped, high abundance (e.g. rRNA, polyA,mtRNA) and unmapped reads. **(B)** Number of identified known miRNAs with transcripts per million (TPM) normalized numbers of counts >1 (blue bars) or >10 (red bars).

All samples passed the post-sequencing QC, which confirmed that the average read quality and base quality had a Q-score > 30 (99.9% correct) (30) and the expected read length distribution for miRNAs (**Supplementary Figure 1**). All samples were sequenced in one excellent run with a median 27.2 million read number. Following sequencing and trimming, all reads containing identical insert sequence and UMI sequence (insert-UMI pair) were collapsed into a single read and passed into the analysis pipeline. This allowed for true quantification of the miRNAs by eliminating library amplification bias and a better representation of the RNA molecules in the sample. We obtained an average of 1.8 million collapsed reads for each sample and good miRNA mapping reads with a very dominant miRNA peak in most of the samples, indicating a good sample/data quality (**Supplementary Table 1** and **Supplementary Figure 2**). Overall, we obtained an average genome mapping rate of 46.2% (**Figure 1A**), which are values well within the range for plasma samples. After mapping and counting to relevant entries in mirbase_20, the number of known miRNAs was calculated using TPM to measure expression. We found comparable numbers of identified miRNAs using either TPM > 1 (182 miRNAs) or TPM > 10 (125 miRNAs) (**Figure 1B**). We did not identify any sequences identical to those of known miRNAs in miRBase_20 for other organisms. However, we were able to predict 80 miRNAs based on the structural properties of the genome in the indicated locations resembling those of known miRNAs (**Supplementary Table 2**).

**Figure 2:**
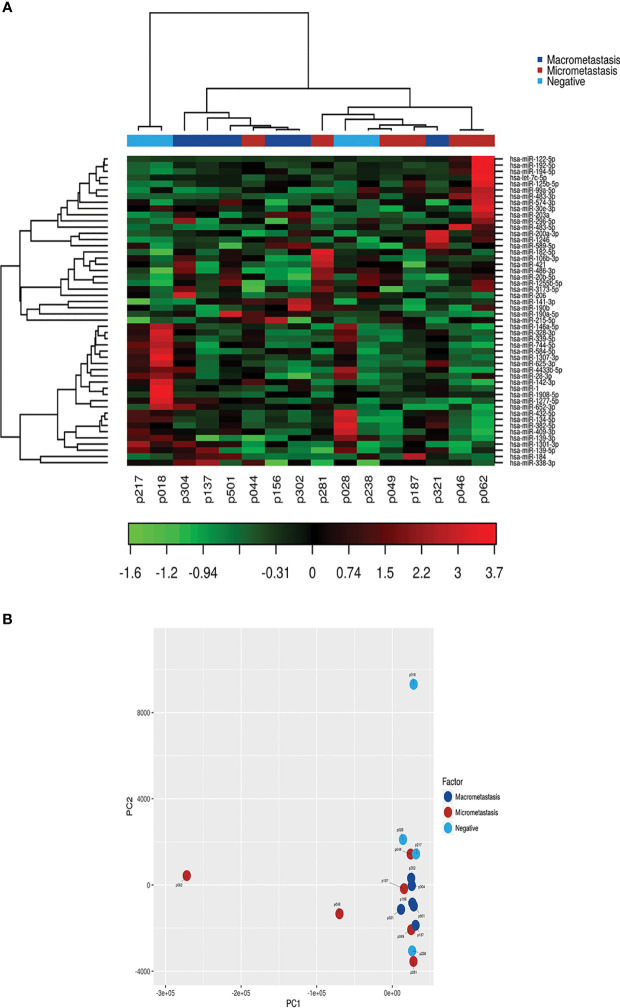
Class discovery associated with the SLN metastatic status. The analysis was performed using the 50 miRNAs with the largest coefficient of variation based on trimmed mean of M-values (TMM counts). **(A)** Heat map and unsupervised hierarchical clustering. Each row represents one miRNA and each column represents one sample. The color represents the relative expression level of a miRNA across all samples. The color scale shows the expression level above (red) or below (green) the mean. **(B)** Principal Component Analysis (PCA) of samples according to the SLN metastatic status of patients.

Next, we investigated whether the patients were assigned into biological groups based on their miRNA expression. We performed an unsupervised two-way hierarchical clustering of miRNAs and samples using the 50 miRNAs with the largest coefficient of variation based on TMM counts (**Figure 2A**). Our results show that samples did not cluster according to the SLN outcome of the patients, suggesting that other clinicopathological factors are responsible for the variation on the samples. We obtained similar results using a principal component analysis. Interestingly, the 2 samples showing the greater variability (p18 and p62) corresponded to those patients whose tumors had a mixed pathological component (IDC and ILC) (**Figure 2B**).

**Figure 3:**
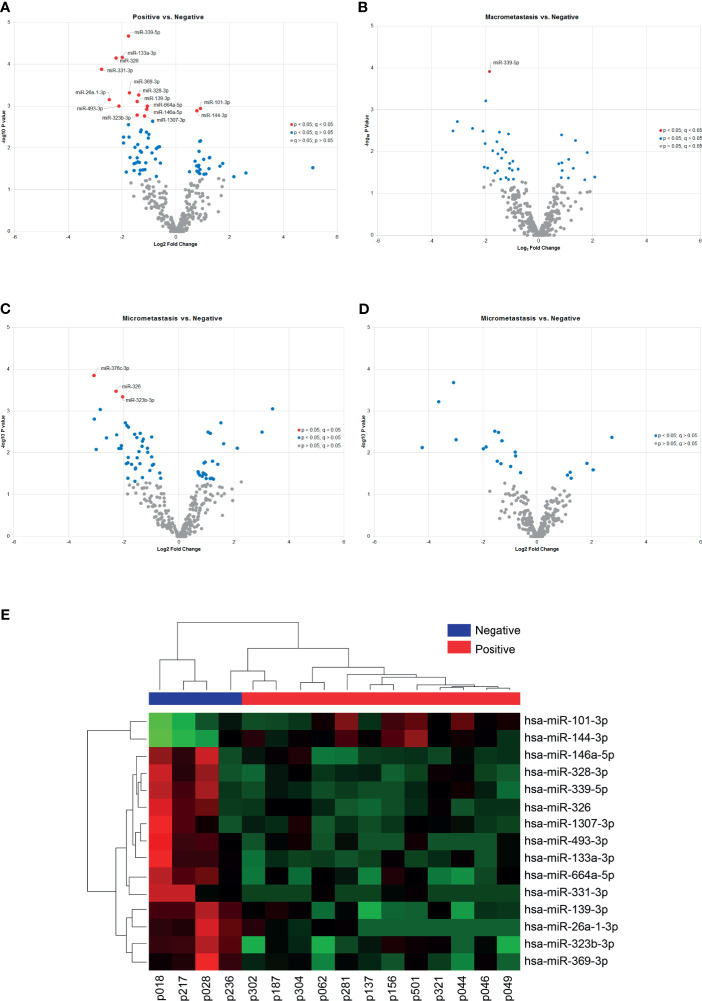
Differentially expressed miRNAs according to the SLN status. **(A–D)** The volcano plots show differentially expressed miRNAs in plasma samples according to the patients’ locoregional metastatic status as indicated. Only significant miRNAs with corrected q values < 0.05 are shown in the plots (red dots). The data show the relationship between non-adjusted p values (y-axis) and the fold change (x-axis) between the experimental groups. **(E)** Heat map and hierarchical clustering analyzed by samples and miRNAs. The analysis was performed using the 15 miRNAs differentially expressed between patients with positive and negative SLNs. Each row represents one miRNA and each column represents one sample. The color scale shows the expression level above (red) or below (green) the mean.

Despite the unsupervised analysis did not group our samples according to the metastasis status of the patients, we identified differentially expressed miRNAs between groups based on the SLN outcome of the patients. First, we analyzed samples according to the positive (n=12) or negative (n=4) SLN metastasis status. We found 73 miRNAs with a significant differential expression (p < 0.05). However, only 16 miRNAs remained significant after correcting for multiple testing (q < 0.05) (**Table 2**). Fourteen miRNAs were down-regulated (miR-339-5p, miR-133a-3p, miR-326, miR-331-3p, miR-369-3p, miR-328-3p, miR-26a-3p, miR-139-3p, miR-493-3p, miR-664a-5p, miR-146a-5p, miR-323b-3pmiR-1307-3p and miR-423-3p) and 2 miRNAs were up-regulated (miR-101-3p and miR-144-3p) (**Figure 3A**). Next, we analyzed the data based on SLN metastasis status subgroups. When we compared patients with macrometastasis vs. negative SLNs, we found 42 miRNAs differentially expressed, but only miR-339-5p remained significant after FDR adjustment (p <0.0001, q = 0.0413) (**Figure 3B** and **Table 2**). Similar results were obtained when we compared micrometastasis and negative SLNs, which yield 66 miRNAs differentially expressed, but only miR-376c-3p (p = 0.0001, q = 0.046), miR-326 (p = 0.0003, q = 0.049) and miR-323b-3p (p = 0.0004, q = 0.049) passed the FDR (**Figure 3C** and **Table 2**). Interestingly, we did not find any significantly differentially expressed circulating miRNAs between patients with macrometastasis or micrometastasis SLNs (**Figure 3D** and **Table 2**). In addition, we used the 16 significant differentially expressed miRNAs in patients with positive SLNs to build a heatmap and hierarchical clustering. Our results show that these miRNAs clearly separated patients with negative and positive SLNs (**Figure 3E**). Further validation on the same clinical samples was performed by specific qPCR assays. The down-regulation of 9 out of 14 miRNAs was confirmed in patients with positive SLNs, but we could not validate the up-regulation of miR-101-3p and miR-144-3p. Furthermore, the degree of down-regulation was higher for those patients that had additional metastases in their axillar lymph nodes (**Supplementary Figure 3**).

**Table 2:** Differentially expressed miRNAs.

Names	Sequence (5’ – 3’)	TMM	TMM	logFC	p value	q value
		Positive	Negative			
hsa-miR-339-5p	TCCCTGTCCTCCAGGAGCTCACG	37.4	127.6	-1.8	< 0.0001	0.007
hsa-miR-133a-3p	TTTGGTCCCCTTCAACCAGCTG	6.1	28.4	-2.0	< 0.0001	0.008
hsa-miR-326	CCTCTGGGCCCTTCCTCCAG	10.6	50.0	-2.2	< 0.0001	0.008
hsa-miR-331-3p	GCCCCTGGGCCTATCCTAGAA	0.9	10.3	-2.8	0.0001	0.011
hsa-miR-369-3p	AATAATACATGGTTGATCTTT	7.9	26.0	-1.7	0.0005	0.031
hsa-miR-328-3p	CTGGCCCTCTCTGCCCTTCCGT	134.1	350.9	-1.4	0.0005	0.031
hsa-miR-26a-1-3p	CCTATTCTTGGTTACTTGCACG	2.1	10.9	-2.5	0.0007	0.034
hsa-miR-139-3p	TGGAGACGCGGCCCTGTTGGAGT	30.2	79.8	-1.4	0.0008	0.034
hsa-miR-493-3p	TGAAGGTCTACTGTGTGCCAGG	1.9	11.5	-2.1	0.0010	0.034
hsa-miR-664a-5p	ACTGGCTAGGGAAAATGATTGGAT	49.7	108.1	-1.1	0.0010	0.034
hsa-miR-101-3p	TACAGTACTGTGATAACTGAA	6070.1	3215.4	0.9	0.0011	0.034
hsa-miR-146a-5p	TGAGAACTGAATTCCATGGGTT	2960.5	6266.3	-1.1	0.0012	0.034
hsa-miR-144-3p	TACAGTATAGATGATGTACT	511.3	295.1	0.8	0.0013	0.035
hsa-miR-323b-3p	CCCAATACACGGTCGACCTCTT	10.5	29.9	-1.4	0.0016	0.040
hsa-miR-1307-3p	ACTCGGCGTGGCGTCGGTCGTG	150.3	337.4	-1.2	0.0017	0.040
hsa-miR-423-3p	AGCTCGGTCTGAGGCCCCTCAGT	353.7	649.1	-0.9	0.0023	0.050
hsa-miR-376c-3p	AACATAGAGGAAATTCCACGT	3.8	14.0	-1.8	0.0028	0.056
hsa-miR-1	TGGAATGTAAAGAAGTATGTAT	69.5	168.7	-1.3	0.0037	0.071
hsa-miR-1908-5p	CGGCGGGGACGGCGATTGGTC	23.1	59.1	-1.3	0.0042	0.073
hsa-miR-744-5p	TGCGGGGCTAGGGCTAACAGCA	138.5	298.1	-1.1	0.0042	0.073
hsa-miR-584-5p	TTATGGTTTGCCTGGGACTGAG	419.9	835.4	-1.0	0.0048	0.078
hsa-miR-6721-5p	TGGGCAGGGGCTTATTGTAGGAG	2.4	9.3	-1.9	0.0055	0.083
hsa-miR-432-5p	TCTTGGAGTAGGTCATTGGGTGG	130.1	432.4	-1.7	0.0055	0.083
hsa-miR-28-3p	CACTAGATTGTGAGCTCCTGGA	72.1	150.4	-1.0	0.0058	0.084
hsa-miR-29b-3p	TAGCACCATTTGAAATCAGTGTT	292	154	0.93	0.0068	0.094
	**Macrometastasis**		**Negative**			
hsa-miR-339-5p	TCCCTGTCCTCCAGGAGCTCACG	38.9	138.0	-1.8	0.0001	0.041
	**Micrometastasis**		**Negative**			
hsa-miR-376c-3p	AACATAGAGGAAATTCCACGT	1.5	13.7	-3.1	0.0001	0.046
hsa-miR-326	CCTCTGGGCCCTTCCTCCAG	9.8	49.2	-2.3	0.0003	0.049
hsa-miR-323b-3p	CCCAATACACGGTCGACCTCTT	7.1	29.5	-2.0	0.0004	0.049
	**Macrometastasis**		**Micrometastasis**			
hsa-miR-122-5p	TGGAGTGTGACAATGGTGTTTG	8948.2	76103.3	-3.1	0.0002	0.062
hsa-miR-125b-2-3p	TCACAAGTCAGGCTCTTGGGAC	0.3	8.9	-3.6	0.0006	0.090

Data shows the 25 most significant differentially expressed miRNAs according to the metastatic status of patients. The list includes the average trimmed mean of M-values (TMM) values, logarithmic fold change (logFC), raw p values and Benjamini-Hochberg FDR corrected q values.

**Figure 4:**
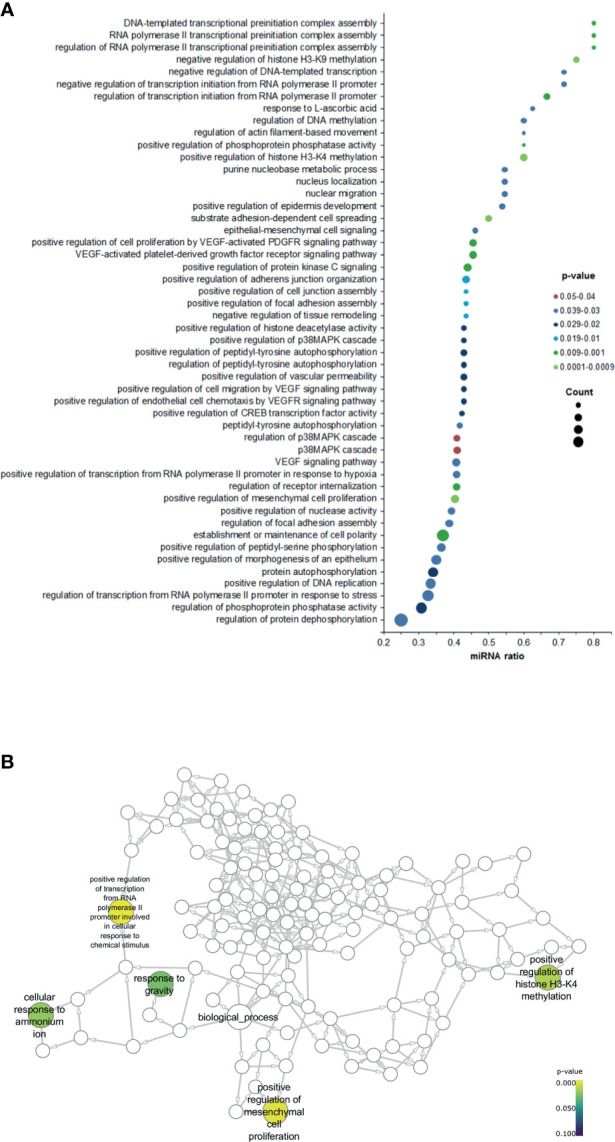
Gene ontology (GO) enrichment analysis for the significant biological processes associated with positive SLNs. **(A)** Dot plot graph shows the 50 most significant biological process GO terms (y-axis) and the ratio between the number of expressed miRNAs associated to the GO term and the number of significantly differentially expressed genes associated to the GO term (x-axis). The color of the nodes indicates the p-value and the size of the nodes the number of miRNAs associated with a specific GO term. **(B)** Neural network shows the GO terms for the biological processes associated with patients with positive SLNs.

Next, we sought to understand how our data is related to biological functions by performing a gene ontology (GO) analysis. Selecting *Homo sapiens* as the background of listed target genes, we obtained the GO term annotations and KEGG pathway analysis through the functional annotation summaries. The results are summarized in **Figure 4** and **Table 3**. The top 50 biological process GO terms (p < 0.05) associated with differentially expressed circulating miRNAs in patients with positive SLNs compared to the reference background (negative SLNs samples) are shown in **Figure 4A**. Our data shows that differentially expressed miRNAs associated with biological processes (BP) markedly focused on epigenetic gene expression regulation, the epithelial-mesenchymal transition (EMT), transcription, cell motility and proliferation processes (p < 0.01) (**Figure 4B**, **Table 3** and **Supplementary Data**). For instance, we found that positive regulation of mesenchymal cell proliferation term (GO: 0002053) was significantly enriched (p < 0.0028) as well as positive regulation of the histone H3-H4 methylation term (GO:0051571) (p < 0.0017). These two GO terms remained significant even when patients with positive SLNs were sub-classified as having macro- or micrometastasis in their SLNs. As for the cellular component (CC), the target miRNAs were significantly located in vesicle and membrane fractions (p < 0.01). Moreover, differentially expressed miRNAs were enriched in molecular function (MF) terms associated with transcription factors, G protein-related coupled peptide receptor activity, receptor regulator activity and microtubule motor activity (p < 0.01) (**Table 3**).

**Table 3:** Gene Ontology (GO) analysis.

GO ID	GO Term	Counts	p value
BIOLOGICAL PROCESS			
GO:0051571	positive regulation of histone H3-K4 methylation	9/15	0.0017
GO:0042462	eye photoreceptor cell development	12/26	0.0017
GO:0070555	response to interleukin-1	17/40	0.0021
GO:0002053	positive regulation of mesenchymal cell proliferation	25/62	0.0028
GO:0071320	cellular response to cAMP	12/23	0.0034
GO:0002407	dendritic cell chemotaxis	4/5	0.0035
GO:0009629	response to gravity	6/8	0.0042
GO:0007097	nuclear migration	6/9	0.006
GO:0051573	negative regulation of histone H3-K9 methylation	6/8	0.0064
GO:0034446	substrate adhesion-dependent cell spreading	8/16	0.0067
**CELLULAR COMPONENT**			
GO:0031091	platelet alpha granule	18/44	0.0065
GO:0031983	vesicle lumen	17/46	0.0137
GO:0060205	cytoplasmic membrane-bounded vesicle lumen	17/46	0.0137
GO:0031093	platelet alpha granule lumen	16/41	0.0171
GO:0034774	secretory granule lumen	16/41	0.0171
GO:0044306	neuron projection terminus	9/20	0.0216
GO:1902495	transmembrane transporter complex	14/31	0.0218
GO:1990351	transporter complex	14/31	0.0218
GO:0015030	Cajal body	8/16	0.0239
GO:0034704	calcium channel complex	7/12	0.0245
**MOLECULAR FUNCTION**			
GO:0008528	G-protein coupled peptide receptor activity	9/15	0.0013
GO:0001618	virus receptor activity	6/13	0.0018
GO:0030955	potassium ion binding	5/6	0.0064
GO:0005161	platelet-derived growth factor receptor binding	17/39	0.0078
GO:0008798	beta-aspartyl-peptidase activity	4/7	0.0097
GO:0030545	receptor regulator activity	15/36	0.0097
GO:0003777	microtubule motor activity	11/20	0.0115
GO:0046625	sphingolipid binding	5/6	0.0128
GO:0008307	structural constituent of muscle	6/8	0.0131
GO:0017022	myosin binding	6/11	0.0144

Gene set enrichment analysis using GO categories (biological process, cellular component, molecular function) was applied to extract biological meaning from the identified differentially expressed transcripts and predicted mRNA targets. The top 10 GO categories associated with differentially expressed circulating miRNAs in patients with positive SLNs are shown. Counts refers to the ratio between the number of enriched differentially expressed miRNAs and the total number of miRNAs assigned to these terms. P values were calculated with a combination of the Elim method and the Fisher's exact method. GO terms with p values < 0.05 were considered enriched.

Our series include 16 patients with early breast cancer and we reported recurrence in 3 (19%) patients. The median follow-up time was 5.2 years (range 2.2 - 6.4 years). Two patients had secondary tumors in the colon and 1 patient in the liver. At last follow-up, two patients with recurrences in colon were reported alive with disease and we reported 3 deaths in patients due to complications related to the disease. We investigated whether the differential expressed miRNAs correlated with the patient’s clinico-pathological parameters. The expression of miR-326, miR-26a-1-3p, miR-139-3p, miR-101-3p, miR-146a-5p and miR-144-3p was significantly lower associated in younger patients (< 60 years, p < 0.05), the expression of miR-328-3p and miR-144-3p was associated with further metastases in the aLNs (p < 0.05), miR-26a-1-3p,miR-144-3p and miR-323-3p were associated with tumor stage (p < 0.05), miR-664a-5p and miR-323b-3p showed a non-significant association with tumor status (p = 0.077 and p = 0.069, respectively) and miR-26a-1-3p showed a non-significant correlation with the recurrence status (p = 0.067). We did not find any other significant association with other parameters. Due to the low number of events, we were unable to perform any survival analysis in our cohort of patients.

## Discussion

In recent years, miRNAs have emerged as important regulators of the various steps of the metastatic process (41). Currently, lymph node affection remains the most important prognosis factor in breast cancer (2) and the presence of metastasis in the SLNs is still currently the recommended procedure for axillary staging of early breast cancer. The accurate evaluation of patients with involved SLN determines further axillary lymph node dissection (ALND), the golden standard procedure for invasive breast cancer. However, ALND has been questioned in recent years because of inherent morbidity following the procedure without directly contributing to survival. In this study, we sought to gain a better understanding of the role of miRNAs in the metastatic process and whether specific expression patterns of miRNAs could predict SLN metastatic status in patients with early breast cancer. We performed a proof-of-principle study in plasma samples from 16 breast cancer patients with known SLN metastasis status. Importantly, plasma samples were collected prior to any treatment, thus the results using RNA-sequencing reflect the basal miRNA expression prior to any therapeutic intervention in these patients. Our results show a good quality sequencing data with mapping rates to miRNAs and comparable miRNA discovery across samples. Thus we are confident in the accuracy of the reported results.

Our data shows that 16 miRNAs were significantly differentially expressed in plasma samples from SLN-positive patients. Overall, we found a general down-regulation of miRNAs, with the exception of miR-101-3p and miR-144-3p that showed a 1.9- and 1.7-fold change up-regulation, respectively. However, we could not confirm the up-regulation of these 2 miRNAs and these results agree with the discrepancies on the direction of the dysregulation for both miRNAs. For instance, dysregulation miR-101-3p has been reported in several malignancies, including breast cancer (42, 43). While some reports indicate up-regulation of miR-101-3p, others indicated the opposite (42). This due to the fact that mature miR-101-3p originates from two different precursors located at different chromosomes. One precursor may be processed to 1 or 2 miRNAs and thus, the mature and precursor miRNA levels might not correlate, and this therefore will influence the clinical interpretation. The same study looked at putative miR-101-3p target genes were analyzed and the most predominant functions were transcription, metabolism, biosynthesis, proliferation, and transcription factor binding. This result indicated that candidate genes have a definitive impact on the pathogenesis of BC (42). Similar conflicting data has been reported for miR-144-3p. In several human cancers, the expression of miR-144-3p has been shown decreased (44), but in animal models repression of miR-144 significantly decreased cell proliferation, clonogenicity, migration and tumor formation in nude mice (45). Interestingly, one report has shown that up-regulation of miR-144-3p was associated with families at high-risk for breast cancer (46). These data suggest that the role of miR-144-3p might differ by cancer type and tumor microenvironment.

Of those miRNAs down-regulated, miR-339-5p showed a 3.5-fold inhibition in patients with positive SLN metastasis. The expression of miR-339-5p remained significant when the analysis was performed in the subgroup of macrometastatic SLNs and we observed a non-significant trend towards significance for the subgroup of micrometastatic SLNs (q = 0.071). Our results agree with previous reports showing that reduced miR-339-5p expression in breast cancer is associated with increased metastasis to lymph nodes (47, 48), high clinical stages and worse clinical outcome (47). A similar association with positive LN has been reported in NSCLC patients (49). In addition, miR-339-5p expression is down-regulated in several human cancers including NSCLC (49), ovarian carcinoma (50), hepatocellular carcinoma (51), gliomas (52), colorectal cancer (53), osteosarcoma (54) and breast cancer (48). Mir-339-5p acts as a tumor suppressor gene and its expression is required to inhibit cell migration and invasion in breast cancer cells (47) in a mechanism that involves at least the B-cell lymphoma 6 (BCL6) protein. The authors showed that forced expression of BCL6 results in increased proliferation, anchorage-independent growth, migration, invasion and survival of breast cancer cell lines, whereas knockdown of BCL6 expression reduced these oncogenic properties of breast cancer cells (55). Interestingly, miR-339-5p has been shown to inhibit migration and invasion by targeting BCL6 in breast cancer (56), ovarian cancer cell lines (50) and in NSCLC (57). In addition, miR-339-5p down-regulation in NSCLC inhibits metastasis of NSCLC by regulating the epithelial-to-mesenchymal (EMT) transition *via* BCL6 (57). A recent report has shown that miR-339-5p regulates EMT through regulation of TGF-β (58) in osteosarcoma (54).

The EMT and the TGF-β pathways are two of the most important mechanisms underlying the metastatic ability of cancer cells (59, 60). We have previously shown the importance of the EMT in breast cancer (61) and here, we show that GO term analysis based on the DE miRNAs showed a significant association with the biological process “positive regulation of mesenchymal cell proliferation” (GO:0002053). Another pathway enriched was the positive regulation of H3 K4 methylation (GO:0051571), a mark that on a genome-wide scale is broadly associated with transcriptional regulation, and “negative regulation of H3K9 methylation” (GO:0051573). H3K9 methylation has been associated with the EMT through interactions of KDM1A (a H3K9 demethylase) with the members of the SNAI1 family of zinc finger transcription factors, including SNAI1 (SNAIL) and SNAI2 (SLUG). The expression of SNAI1 and E-cadherin is a hallmark of carcinoma development and metastasis (62). Our data suggest that that MiR-101 could be involved in the regulation of these pathways, as it has been shown to directly target the histone methyltransferase enhancer of zeste homologue 2 (EZH2), which could promote tumor proliferation and invasion (63).

Among the other circulating miRNAs that were down-regulated in our study, the expression of miR-133a-3p has been reported to be down-regulated in paired breast cancer tumor and serum samples (64), suggesting the tumor origin of miR-133a-3p. In contrast, miR-133a-3p has been found elevated in plasma samples from early-stage BC patients compared to healthy donors (65, 66) and similar results have been reported for circulating miR-1307-3p (67). On the other hand, down-regulation of miR-376c-3p (68) and miR-376c-3p have been linked to breast cancer recurrence (24). MiR-326 has been reported to target B7-H3 in breast cancer, an immunoregulatory protein that is overexpressed in several cancers and is often associated with metastasis and poor prognosis (69). Furthermore, its expression has been shown to inhibit tumorigenesis through direct targeting of Nin one binding protein (NOB1) and the MAPK pathway in glioma cells (52).

A main limitation of our study is the small number of samples analyzed since it was designed as a proof-of-principle study to assess the feasibility of using circulating miRNAs as potential surrogates of the lymph node metastatic status in breast cancer. Therefore, our results are preliminary and must be interpret with caution. Nonetheless, our data shows several circulating miRNAs that are significantly differentially expressed in relation to the SLN metastatic status of the patients. Moreover, we report an overall down-regulation of these miRNAs, which in most cases have been reported to be direct targets of proteins that promote metastasis. Further studies in a larger cohort of patients are warranted to validate these results and to unveil the molecular mechanisms of the miRNAs described here and the various steps of the locoregional metastasis.

## Data Availability Statement

The original contributions presented in the study are publicly available at the Sequence Research Archive under ID PRJNA669408 and are available for download here http://www.ncbi.nlm.nih.gov/bioproject/669408.

## Ethics Statement

This study was conducted according to the Declaration of Helsinki principles, with approval from the Clinical Research Ethics Committee at “Institut d’Investigació’ Biomédica Sant Pau” (IIB Sant Pau). Written informed consent was obtained from all patients under institutional review board-approved protocols. All methods were performed in accordance with the relevant guidelines and regulations.

## Author Contributions

DE, LL-V, and AB were involved in the conceptualization of the project. DE, LL-V, OB, JM, IP, AM, CA, BGV, TR and EL were involved in resources, investigation and methodology. DE, LL-V, EL, and AB were involved in analysis and interpretation of data. All authors contributed to the article and approved the submitted version.

## Funding

Instituto de Salud Carlos III and European Regional Development Fund (PI13/00110 and PI19/00362 to D.E.); Centro de Investigación Biomédica en Red Cáncer (CB 16/12/00471 to AB); Red Temática de Investigación Cooperativa de Cáncer (RTICC; RD12/0036/0076 to all authors.

## Conflict of Interest

The authors declare that the research was conducted in the absence of any commercial or financial relationships that could be construed as a potential conflict of interest.

## Publisher’s Note

All claims expressed in this article are solely those of the authors and do not necessarily represent those of their affiliated organizations, or those of the publisher, the editors and the reviewers. Any product that may be evaluated in this article, or claim that may be made by its manufacturer, is not guaranteed or endorsed by the publisher.

## Supplementary Material

Supplementary Figure 1 Data quality checking. **(A)** Average read quality of the next generation sequencing (NGS) data. The average read Q-score is plotted on the x-axis and the number of reads on the y-axis. A Q-score above 30 is considered high quality data. **(B)** Base quality of the NGS data. The position in the read is plotted on the x-axis and the Q-score is plotted on the y-axis. The red line is the median Q-score and the dark blue is the mean value Q-score. A Q-score above 30 (>99.9% correct) is considered high quality data. **(C)** Read length distribution and adaptor trimming. miRNAs will appear as a peak around 18-23 nucleotides. **(D)** Radar plot showing relative spike-in signal for the samples. A range of spike-ins was added to the samples prior to RNA isolation. We observed an excellent correlation of counts corresponding to the spike-ins between the samples.

Supplementary Figure 2 Summary of the mapping results for the samples. **(A)** Total number of reads for each sample sequenced. **(B)** Read length distribution for each class of RNAs. The peak around 18-23 nucleotides (red) corresponds to miRNAs.

Supplementary Figure 3 RNA-sequencing validation. Relative gene expression was performed according to the comparative ddCt (ΔΔCt) method using negative metastatic samples as reference. The geNorm or the Normfinder algorithm were used to select the best combination of two reference genes. Data from multiples plates were normalized using UniSp3 spike-in as interplate calibrators. Each microRNA was assayed twice by qPCR on the Serum/plasma Focus microRNA PCR panel. **(A)** Data shows the comparison between patients with negative and positive SLNs. **(B)** Patients with positive SLNs were divided according to the presence or absence of further axillary lymph nodes (aLNs). Statistical analysis was performed using unpaired Student’s t-test. All comparisons shown are statistically significant (p < 0.05).

Supplementary Table 1 Mapping and yield results of the sequencing data. Reads are classified into the following classes: miRNA, maps to mirBase_20; small RNA, maps to sRNA database compiled by Exiqon; Predicted miRNA, predicted miRNAs using a prediction software; Genome, reads aligning to the reference genome, but not to miRNAs or sRNAs; Outmapped, maps corresponding to poly A and poly homopolymers as well as abundant rRNA and mtRNA; Unmapped, reads not aligning to reference genome. Median values for each type of reads are shown.

Supplementary Table 2 Predicted miRNA results of the sequencing data. Predicted miRNAs for each sample sorted by total read count. The sequences reported do not match any known miRNA in the miRBase_20. The location is formatted as "chromosome: start-stop (strand)". Counts describes the number of reads which fall onto the location of the predicted miRNA (sum of all samples). Note that the name and numbering assigned here are project-specific and cannot be compared with the nomenclature found in the miRBase or other databases.

Supplementary Table 3 Contributing miRNAs to GO terms for the categories biological process (BP), molecular function (MF) and cellular compartment (CC).
