# Supplementary figures and images for: Circulating microRNAs in Early Breast Cancer Patients and Its Association With Lymph Node Metastases
Source: Front Oncol. 2021 Aug 26;11:627811. doi: 10.3389/fonc.2021.627811 (PMC8428362; doi:10.3389/fonc.2021.627811)

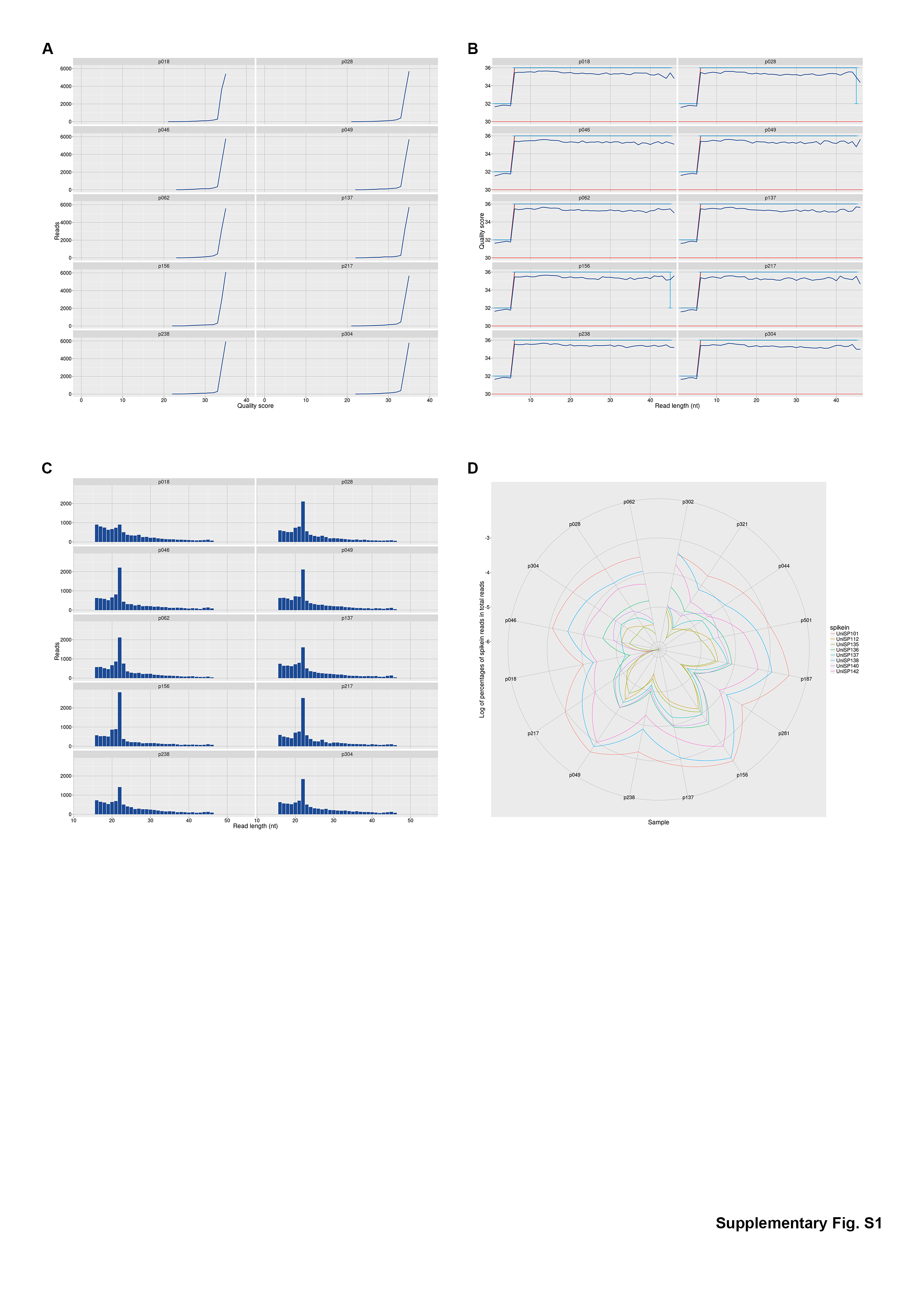

Supplement: Supplementary Figure 1 — Data quality checking. (A) Average read quality of the next generation sequencing (NGS) data. The average read Q-score is plotted on the x-axis and the number of reads on the y-axis. A Q-score above 30 is considered high quality data. (B) Base quality of the NGS data. The position in the read is plotted on the x-axis and the Q-score is plotted on the y-axis. The red line is the median Q-score and the dark blue is the mean value Q-score. A Q-score above 30 (>99.9% correct) is considered high quality data. (C) Read length distribution and adaptor trimming. miRNAs will appear as a peak around 18-23 nucleotides. (D) Radar plot showing relative spike-in signal for the samples. A range of spike-ins was added to the samples prior to RNA isolation. We observed an excellent correlation of counts corresponding to the spike-ins between the samples. [file Image_1.tif]

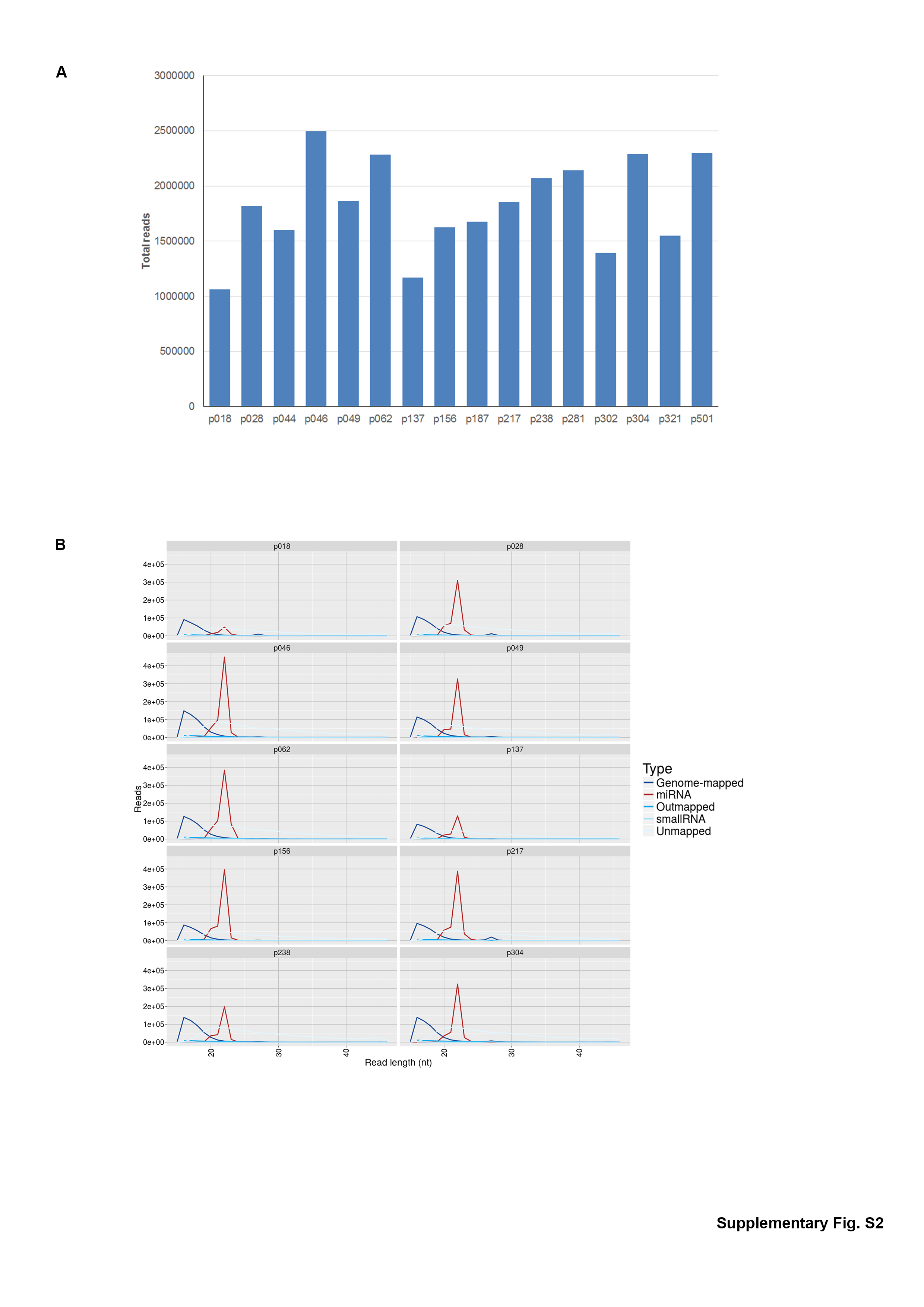

Supplement: Supplementary Figure 2 — Summary of the mapping results for the samples. (A) Total number of reads for each sample sequenced. (B) Read length distribution for each class of RNAs. The peak around 18-23 nucleotides (red) corresponds to miRNAs. [file Image_2.tif]

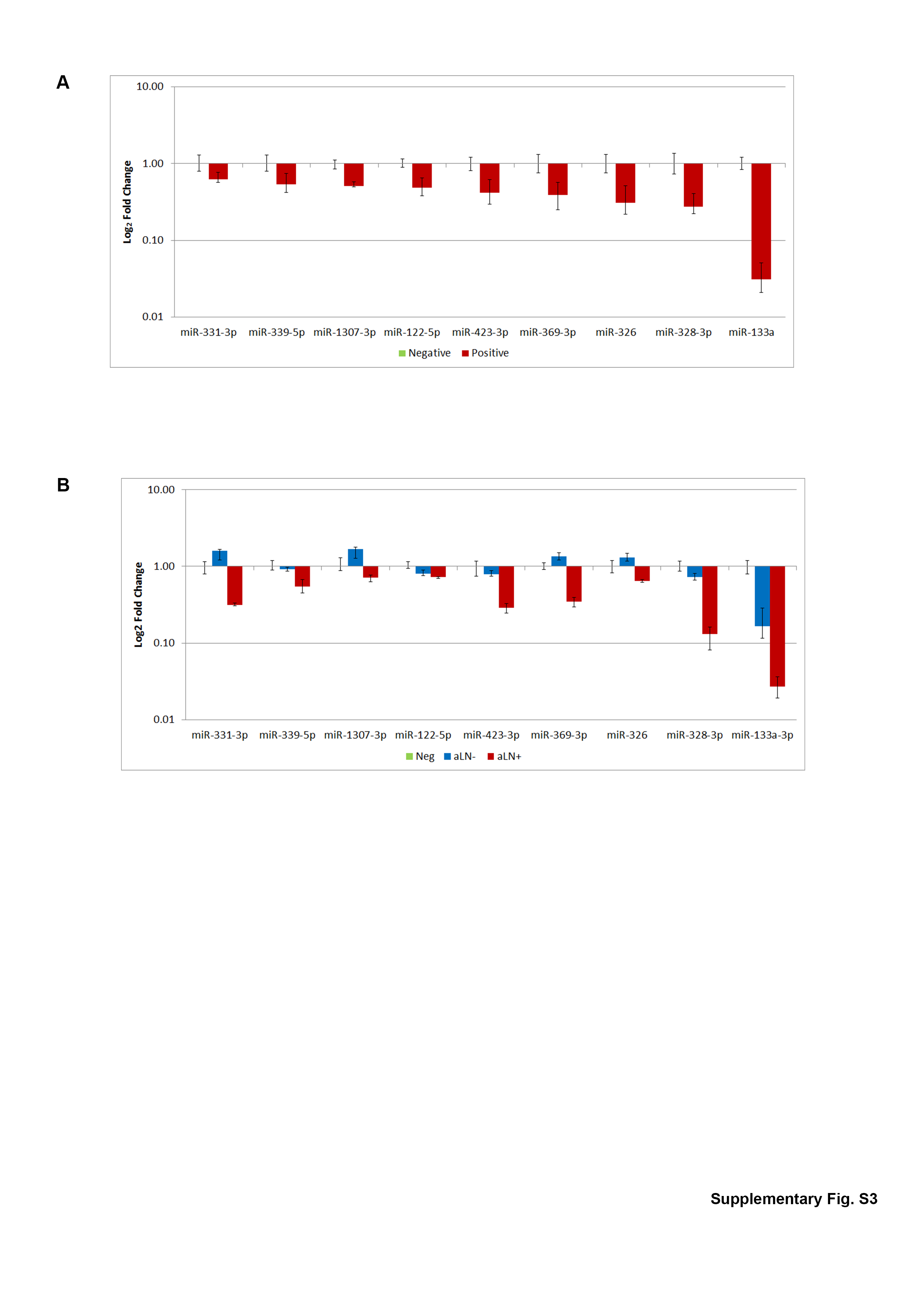

Supplement: Supplementary Figure 3 — RNA-sequencing validation. Relative gene expression was performed according to the comparative ddCt (ΔΔCt) method using negative metastatic samples as reference. The geNorm or the Normfinder algorithm were used to select the best combination of two reference genes. Data from multiples plates were normalized using UniSp3 spike-in as interplate calibrators. Each microRNA was assayed twice by qPCR on the Serum/plasma Focus microRNA PCR panel. (A) Data shows the comparison between patients with negative and positive SLNs. (B) Patients with positive SLNs were divided according to the presence or absence of further axillary lymph nodes (aLNs). Statistical analysis was performed using unpaired Student’s t-test. All comparisons shown are statistically significant (p < 0.05). [file Image_3.tif]
